# Comprehensive protocol for preparing diatom cell samples and associated bacterial consortia for scanning electron microscopy

**DOI:** 10.1016/j.xpro.2024.103380

**Published:** 2024-11-01

**Authors:** Margaux Maillard, Nicolas Stephant, Atsuko Tanaka, Leïla Tirichine

**Affiliations:** 1 Nantes Université, CNRS, US2B, UMR 6286, 44000 Nantes, France; 2 Nantes Université, CNRS, Institut des Matériaux de Nantes Jean Rouxel, IMN, 44000 Nantes, France; 3 Department of Chemistry, Biology and Marine Science, Faculty of Science, University of the Ryukyus, Okinawa 903-0213, Japan; 4 Institute for Marine and Antarctic Studies (IMAS), Ecology and Biodiversity Centre, University of Tasmania, Hobart, TAS 7004, Australia

**Keywords:** Microbiology, Microscopy, Model Organisms

## Abstract

Meticulous sample preparation and strict adherence to preservation procedures are essential for electron microscopy investigations, which enable accurate capture of organisms’ morphology, size, and potential interactions within the sample. Here, we present a protocol for preserving cells of the model diatom *Phaeodactylum tricornutum* and its native bacterial community. We describe steps for diatom fixation and coverslip preparation and washing. We then detail procedures for dehydrating, drying, and metallizing samples followed by observation using scanning electron microscopy.

## Highlights

• Detailed fixation procedure for samples using PHEM buffer
• Instructions for preparing poly-L-lysine-coated coverslips for sample adherence
• Steps for drying samples by gradually replacing absolute ethanol with liquid CO_2_

## Abstract (editor highlights)

Publisher’s note: Undertaking any experimental protocol requires adherence to local institutional guidelines for laboratory safety and ethics.

## Graphical abstract

**Figure:**
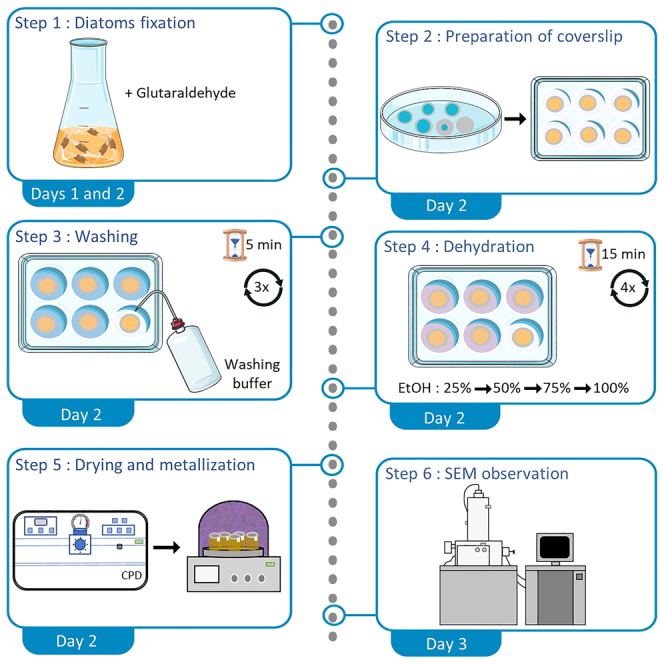

## Before you begin

Diatoms, a crucial group of eukaryotic phytoplankton, are distributed across all latitudes in marine and freshwater ecosystems. They play a fundamental role in the biogeochemical cycling of elements such as carbon and silicon.^1^^,^^2^^,^^3^ They are responsible for around 45% of global primary production and yet represent only 1% of Earth’s photosynthetic biomass.^4^ In addition to their ecological importance, diatoms find widespread use in nanotechnology, aquaculture and in the pharmaceutical and industrial sectors.^5^^,^^6^

Diatoms do not live in isolation; they have coexisted with prokaryotes since early evolutionary stages. These interactions are critical for nutrient fluxes and the cycling of elements such as iron, nitrogen, vitamins, and phosphorus.^3^ Bacteria are crucial to microalgae as they supply essential components like vitamins, growth factors, iron and nitrogen among other benefits. In return, microalgae produce organic compounds through photosynthesis, which bacteria use as a carbon source. Both microalgae and bacteria enhance each other’s resilience to environmental conditions. Bacteria alter the microenvironment to make it more suitable for algal growth, while microalgae form biofilms that provide protection and facilitate nutrient exchange.^7^^,^^8^ Therefore, investigating these interactions at the electron microscopic scale will provide critical insights into their mechanisms, thereby advancing and complementing the knowledge acquired through other methods. Despite their significance in contributing to primary productivity at the base of the food chain, the nature of these interactions at the microscopic, specifically nanometer scale remains poorly understood. This knowledge gap is primarily due to the challenges in handling microscopic samples, particularly in liquid media. Additionally, technologies like electron microscopy, which provide microscopic-scale access, require rigorous sample preparation since the quality of the sample directly affects the output and subsequent information and data extraction. Our protocol is simpler and requires fewer steps compared to previously described methods. It is suitable for both microalgae and their associated bacterial consortia, which previous protocols do not address.^9^^,^^10^ To the best of our knowledge, there are currently no straightforward sample preparation protocols for scanning electron microscopy that effectively capture both eukaryotic and prokaryotic cells while preserving both entities and any potential interactions between them.

The following protocol provides a step-by-step procedure for preparing a sample containing diatoms and their associated bacterial consortia for observation under a scanning electron microscope (SEM). This protocol aims to preserve both the diatoms and the bacteria, as samples are often susceptible to damage during fixation, centrifugation, and dehydration steps if a compromise in the conditions of these steps is not achieved to preserve both entities. An important factor to consider is the starting cell density and centrifugation time and speed to avoid inducing mechanical stress that could damage the cells.

Prior to the fixation step, it is crucial to prepare diatom cells to ensure they are in a healthy exponential growth phase. The duration needed to reach this phase varies between diatom species, depending on their division rate and the initial concentration used to start the culture. For the model diatom used in this study, *Phaeodactylum tricornutum* accession 11,^11^^,^^12^ one week was sufficient to reach the exponential phase, achieving a concentration of 9 million cells/mL from an initial concentration of 100,000 cells/mL. Both PHEM^13^ (PIPES, HEPES, EGTA, magnesium sulfate hexahydrate) and washing buffer solutions must be prepared in advance.

### Preparation of diatom culture

**Timing: 1 week**

1. Diatom cell counting.
  a. Take a sample from the diatom stock culture and place 50 μL on a Malassez cell to determine the concentration in cells/mL.
2. Preparing Diatom Culture at 100,000 cells/mL.
  a. Calculate the volume of diatoms needed to inoculate a new culture at 100,000 cells/mL in an Erlenmeyer flask containing Enhanced Artificial Sea Water medium.^14^
  b. Incubate the culture in a chamber at 19°C, shaking at 120 rpm, with a 12:12-h light-dark cycle at 50 μE for 7 days to reach the exponential growth phase of diatoms.

**CRITICAL:** Make sure to use a laminar flow hood when handling diatoms cultures to maintain sterility.

### Preparation of 10X PHEM buffer

**Timing: 30 min**

The PHEM buffer is chosen for its compatibility with most fixatives, including glutaraldehyde. Additionally, it offers robust pH stability across a range of temperatures and conditions, which is crucial for preserving sample integrity.

3. Preparation of 200 mL of 10X PHEM Buffer (Quantities are detailed in 10X PHEM buffer table below).
  a. Dissolve the following products in distilled water in the specified order: EGTA, PIPES, HEPES, and MgCl_2_·6H_2_O. Use a beaker and mix with a stirrer.
  b. Adjust the volume to approximately 185 mL with distilled water.
  c. In a separate beaker, dissolve 5 g of NaOH in 15 mL of distilled water. Add this solution to the beaker containing the buffer mixture to adjust the pH to 7.4.

***Note:*** If the pH remains too alkaline, dissolve an additional 4 g of NaOH in 15 mL of distilled water. Add this solution gradually until the desired pH of 7.4 is achieved. Ensure the final volume is 200 mL.

### Preparation of washing buffer (1.5X PHEM, 9% sucrose)

**Timing: 10 min**

4. Preparation of 200 mL of washing buffer from PHEM buffer.
  a. Mix 18 g of sucrose in 30 mL PHEM buffer with stirring.
  b. Add distilled water to reach a final volume of 200 mL.

### Preparation of fixation buffer

**Timing: 10 min**

5. Preparation of 10 mL (for 2 samples) of fixation buffer from PHEM buffer.
  a. In a falcon tube, prepare 9% sucrose (mix 0.9 g of sucrose with 2 mL of distilled water).
  b. Add 1.5 mL of 10X PHEM buffer.
  c. Add 2 mL of 25% glutaraldehyde.
  d. Adjust the volume to 10 mL with distilled water.

***Note:*** Use a laboratory fume hood when handling glutaraldehyde.

## Key resources table

**Table:** 

REAGENT or RESOURCE	SOURCE	IDENTIFIER
**Biological samples**		

*Phaeodactylum tricornutum*	NCMA	NCBI:txid2850

**Chemicals, peptides, and recombinant proteins**		

EGTA	VWR, France	Cat#0732-50g
PIPES	Sigma-Aldrich, France	Cat#P6757
HEPES	VWR, France	Cat#0511-250G
MgCl_2_·6H_2_O	Sigma-Aldrich/Merck, France	Cat#M9272
NaOH	VWR, France	Cat#28244.295
Sucrose	VWR, USA	Cat#0335-500G
Glutaraldehyde 25%	VWR, France	Cat#87043.230
Poly-L-lysine	Sigma-Aldrich/Merck, France	Cat#P4707
Absolute ethanol	Merck, France	Cat#1.00983.1000
PELCO conductive silver paint, 30 g	Ted Pella, Inc., France	Cat#16062

**Experimental models: Organisms/strains**		

*Phaeodactylum tricornutum*	NCMA	NCBI:txid2850

**Other**		

15 mL Falcon tubes	Starlab, France	Cat#E1415-0800
50 mL Falcon tubes	Starlab, France	Cat#E1450-0200
Pipet tips 1000 μL TipOne	Starlab, France	Cat#S1122-1730-C
Petri dish	VWR, France	Cat#391-0469
24-wells plate	Starlab, CytoOne, France	Cat#CC7672-7524
Round coverslips, glass, Ø10 mm	Ted Pella, Inc., USA	Cat#260368
Specimen mount, Ø12.5 × 10 mm	Ted Pella, Inc., USA	Cat#16232
PELCO Tabs, carbon conductive tabs, 9 mm OD	Ted Pella, Inc., USA	Cat#16084-3
PELCO Conductive Silver 187	Ted Pella, Inc., USA	Cat#16045
Micromesh biopsy cassettes, one compartment	Ted Pella, Inc., USA	Cat#27150-5B
Analytical balance	JOANLAB, China	Cat#FA1204
Magnetic stirrer RH basic	IKA, Germany	Cat#0005019700
Basket centrifuge MEGA STAR 1.6R	VWR, France	Cat#521-2661
Critical point dryer CPD 030	Bal-tec	N/A
JUC 5000 JEOL sputter coater	JEOL, France	N/A
SEM FEG JEOL JSM7600F	JEOL, France	N/A
EVACTRON decontaminator RF plasma cleaner	XEI Scientific, Inc., USA	N/A

## Materials and equipment

**Table:** PHEM Buffer 10X (For 200 mL)

Reagent	Final concentration	Amount
EGTA	100 mM	7.6 g
PIPES	600 mM	36.28 g
HEPES	250 mM	11.92 g
MgCl_2_·6H_2_O	20 mM	0.82 g
NaOH	625 mM	5 g
Distilled water	N/A	s.q. 200 mL
**Total**	**N/A**	**200 mL**

Store at 4°C for up to 6 months.

**CRITICAL:** NaOH is corrosive and can cause severe skin burns. Always handle with gloves, protective goggles and a lab coat.

**Table:** Washing Buffer (For 200 mL)

Reagent	Final concentration	Amount
PHEM Buffer 10X	1.5X	30 mL
Sucrose	9%	18 g
Distilled water	N/A	s.q. 200 mL
**Total**	**N/A**	**200 mL**

Store at 4°C for up to 1 month.

**Table:** Fixation Buffer (For 10 mL)

Reagent	Final concentration	Amount
PHEM 10X	1.5X	1.5 mL
Sucrose	9%	0.9 g
Glutaraldehyde 25%	5%	2 mL
Distilled water	N/A	s.q. 10 mL
**Total**	**N/A**	**10 mL**

Store at 4°C for up to 1 month.

**CRITICAL:** Glutaraldehyde is a Carcinogenic, Mutagenic, Reprotoxic (CMR) substance, corrosive, and hazardous to both health and the environment. Handle it in a laboratory fume hood while wearing gloves, goggles, and a lab coat.

## Step-by-step method details

### Step I: Diatom fixation

**Timing: 1–2 days**

**Figure 1:**
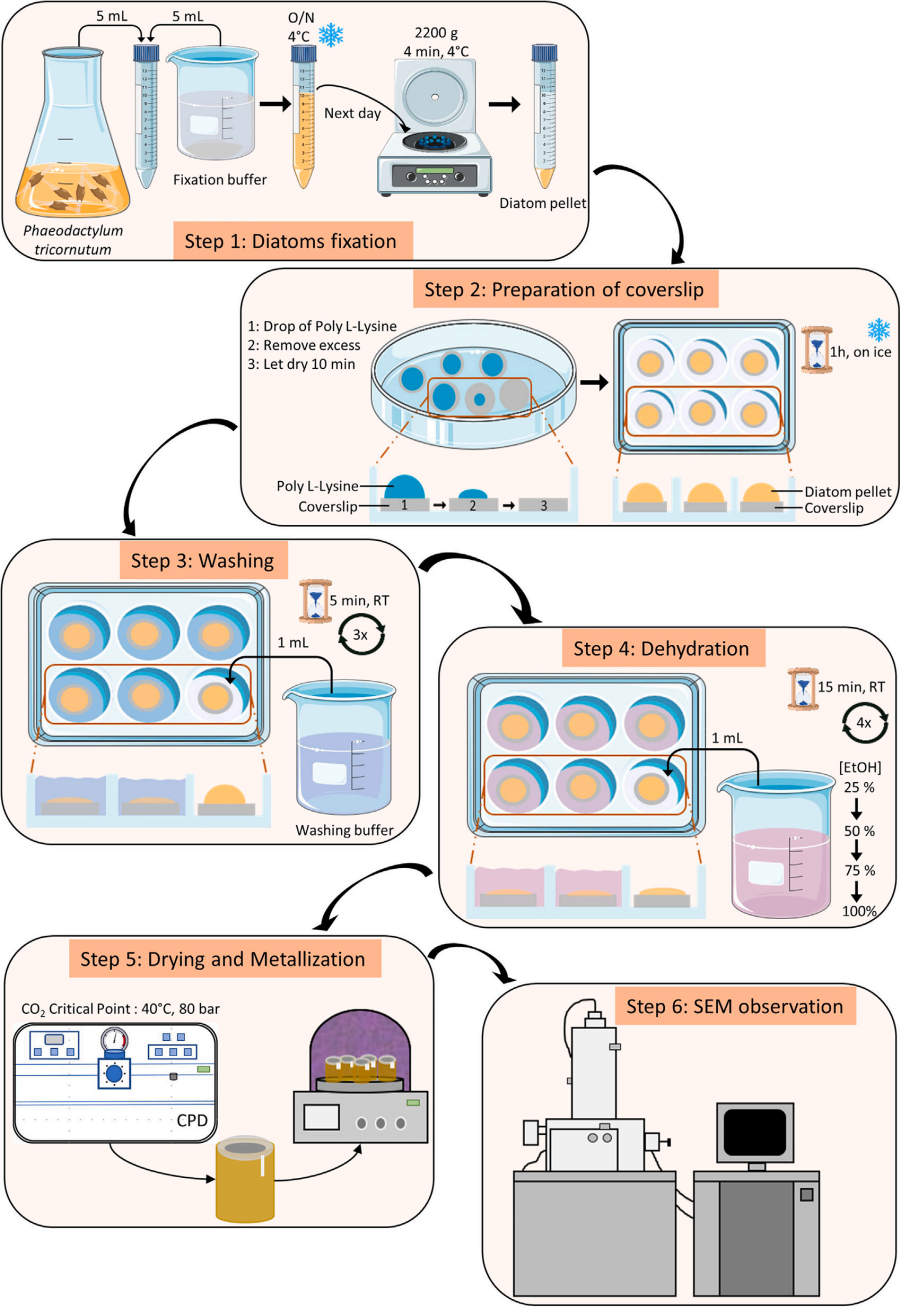
Detailed diagram of the various steps in the protocol: from diatom fixation, through coverslip preparation and mounting, to scanning electron microscope observation RT: Room Temperature.

The aim of this step is to fix diatom cells and associated bacteria while preserving their structures and cellular integrity (Figure 1).

Day 1: 17 h

**Figure 2:**
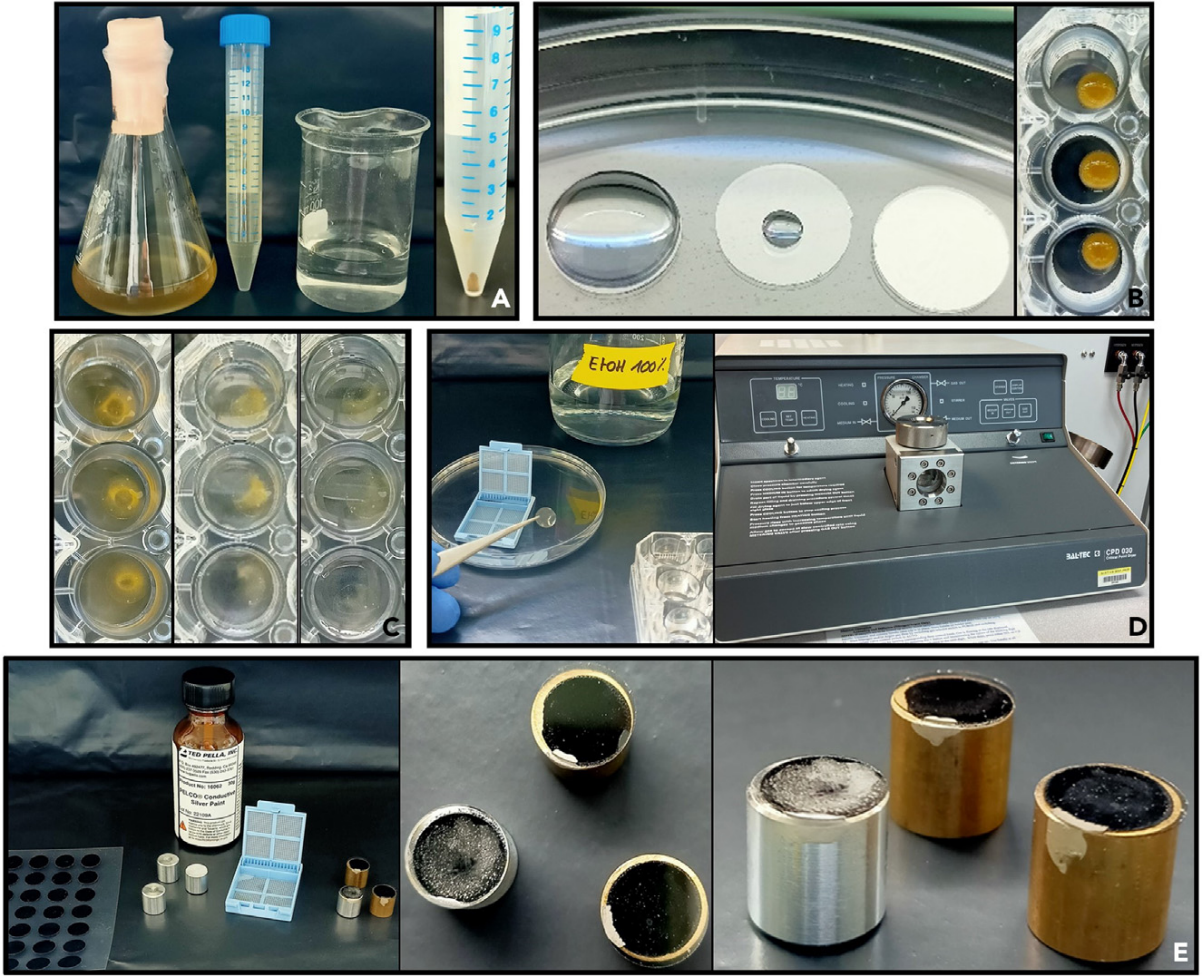
Photographs to illustrate some of the steps in the diatom sample preparation and fixation protocol (A) Fixation of *Phaeodactylum tricornutum* cells by mixing the culture with fixation buffer and pellet formation after the centrifugation (step I). (B) Preparation of coverslips by adding a drop of poly-L-lysine, then removing the excess, allowing it to dry, then adding a drop of the fixed diatom pellet and letting it sediment (step II). (C) Photo of the coverslips during 3 washes with washing buffer (step III). (D) Transfer of coverslips into biopsy cassettes for CO_2_ critical point drying (step V). (E) Mounting of the coverslips on the sample mount with carbon conductive tab and silver paint (step V).

1. Fixation of *Phaeodactylum tricornutum* with fixation buffer (Figure 2A).
  a. Prepare fixation buffer in a 15 mL falcon tube.
  b. Add 5 mL of diatom culture to 5 mL of fixation buffer in a 15 mL falcon tube.
  c. Mix gently by inversion.
  d. Incubate overnight at 4°C.

Day 2: 10 min

2. Concentration of diatom cell by centrifugation (Figure 2A).
  a. Centrifuge the falcon tube at 2200 *g* for 4 min, at 4°C.
  b. Remove most of the supernatant, leaving about 200 μL of supernatant in the falcon tube, to slightly resuspend the diatom pellet.

***Note:*** Use a laboratory fume hood when handling fixation buffer containing glutaraldehyde.

### Step II: Preparation of coverslips

**Timing: 20 min**

The aim of this step is to prepare the coverslips by attaching the diatoms to them using poly-L-lysine, a non-specific cell attachment factor that promotes cell adhesion to solid substrates by enhancing electrostatic interactions.

3. Spreading poly-L-lysine on coverslip (Figure 2B).
  a. Take clean coverslips and place them in an empty petri dish using forceps.
  b. Spread 100 μL of poly-L-lysine solution evenly over the entire surface of the coverslip using a pipette.
  c. Remove excess poly-L-lysine solution by pipetting.
  d. Allow the coverslip to dry for more than 10 min at room temperature.

***Note:*** Remember to close the petri dish lid to prevent dust from settling on the coverslip.

***Note:*** Remember to clearly identify and record the side of the coverslip on which the poly-L-lysine have been deposited.

4. Attachment of diatoms to coverslip (Figure 2B).
  a. Place coverslips in a 24-wells plate, using forceps (1 well = 1 coverslip).
  b. Pipette 100 μL of the diatom pellet onto the coverslip, creating a large drop that covers the entire surface.
  c. Place the plate on ice and allow diatom cells to settle on the coverslip for 1 h.

***Note:*** Use a laboratory fume hood when handling fixation buffer containing glutaraldehyde.

***Note:*** Remember to clearly identify and record the side of the coverslip on which the poly-L-lysine and diatoms have been deposited.

### Step III: Washing

**Timing: 20 min**

The purpose of this step is to wash the coverslip to remove any excess diatoms that have not adhered to it, as well as to eliminate residues of the components comprising the fixation buffer.

5. Gently add 1 mL of washing buffer to each well of the plate (Figure 2C).
  a. Allow to sit for 5 min at room temperature.
  b. Remove the wash buffer with a pipet.
6. Repeat step 5, three times.

***Note:*** Carefully pour the wash buffer against the inner wall of the well. Avoid pouring the wash buffer directly onto the coverslip.

***Note:*** Do not shake the plate to prevent disturbance of the coverslip surface and detachment of the diatoms.

### Step IV: Dehydration

**Timing: 1 h 30 min**

The aim of this step is to dehydrate the samples by gradually replacing the water in the medium with ethanol in increasing concentrations until it is replaced by absolute ethanol.

7. Prepare different concentration of ethanol (25%, 50%, 75%, 100%) in 50 mL falcon tube, from absolute ethanol.
  a. Mix 12.5 mL of absolute ethanol with 37.5 mL of distilled water, to make 50 mL at 25%.
  b. Mix 25 mL of absolute ethanol with 25 mL of distilled water, to make 50 mL at 50%.
  c. Mix 37.5 mL of absolute ethanol with 12.5 mL of distilled water, to make 50 mL at 75%.
  d. Use 50 mL of absolute ethanol, to make 50 mL at 100%.
8. Ethanol bath succession.
  a. Gently, add 1 mL of 25% ethanol to each well containing a coverslip.
  b. Allow to sit 15 min at room temperature.
  c. Remove the solution and replace it with 1 mL of 50% ethanol.
  d. Repeat these steps until 100% ethanol solution is reached.
  e. Make a second bath with 100% ethanol.
  f. Leave coverslips soaked in 100% ethanol until the critical point drying stage.

***Note:*** Carefully pour the ethanol against the inner wall of the well. Avoid pouring it directly onto the coverslip. A 15-min ethanol bath is necessary at each sequential step.

***Note:*** Do not shake the plate to prevent disturbance of the coverslip surface and detachment of the diatoms.

### Step V: Drying and metallization

**Timing: 2 h 30 min**

The aim of this step is to dry the samples by gradually replacing the absolute ethanol with liquid CO_2_, then increasing the pressure and temperature to reach the critical point of CO_2_, transitioning it into a supercritical state. This process dries the samples without subjecting them to high heat, thereby preventing damage. Following this, the coverslip is mounted on a SEM stub and made electrically conductive at the surface by sputter-coating with a 2 nm layer of platinum. This surface is then electrically connected to the stub by a drop of silver paint. If platinum is unavailable, it can be substituted with gold (Au), gold-palladium (Au-Pd), or silver (Ag).

9. Preparing coverslip for drying at the critical point of liquid CO_2_ (Figure 2D).
  a. Place a biopsy cassette inside an empty petri dish.
  b. Fill the petri dish with absolute ethanol until the bottom of the biopsy cassette is covered.
  c. Gently take the coverslip with forceps from one of the plate wells and place it in the biopsy cassette.
  d. Place the biopsy cassette in the critical point dryer chamber and fill it with absolute ethanol until the coverslips and cassettes are submerged.
  e. Close the chamber of the critical point dryer and initiate a drying cycle at the critical CO_2_ point, according to the specifications of the device used. For our device, we used the following steps:
    i. Turn on the CPD and the cooling circuit.
    ii. Open the CO_2_ cylinder and allow the temperature to reach 10°C.
    iii. Fill the CPD chamber with liquid CO_2_, ensuring the level remains below the air bubble at the top of the chamber.
    iv. Activate the stirrer, then drain the chamber down to the level of the coverslip.
    v. Repeat the process of filling and emptying the CPD chamber with liquid CO_2_, 5 to 6 times to completely replace the ethanol with liquid CO_2_ (Approximately 40 min).
    vi. After the final fill, stop the stirrer, ensure all valves are closed, and activate the heating circuit.
    vii. Allow the temperature to reach 40°C, which will increase the pressure inside the chamber to between 80 and 90 bar. Ensure the unit is not damaged by staying within this range (Approximately 10 min).
    viii. Gradually reduce the pressure using the Gas Out button and the metering valve, but do not let it drop below 80 bar to avoid falling below the critical point of CO_2_.
    ix. When the pressure stabilizes and the temperature remains constant, all the CO_2_ has transitioned to its supercritical state (Approximately 15 min).
    x. Once stable, release the pressure by pressing the Gas Out button, setting the Metering valve to 0 bar, and then open the chamber to retrieve the biopsy cassette.

***Note:*** Ensure that you clearly identify and record the side of the coverslip where the diatoms have been deposited.

***Note:*** The coverslip must remain submerged in absolute ethanol at all times between the dehydration and critical point drying stages.

***Note:*** If several samples are to be dried simultaneously, use a scalpel to make a cut in the biopsy cassette to distinguish between them.

10. Mounting coverslips on sample mounts (Figure 2E).
  a. Attach a carbon conductive tab to the top of a sample mount.
  b. Stick coverslip on the carbon conductive tab.
  c. Apply a line of silver paint between the sample mount and the coverslip.

***Note:*** Samples can be identified by adding a number below the specimen mounts.

***Note:*** Pay attention to the orientation of the coverslip. The side with the diatoms attached must be the side visible from the top of the specimen mounts.

11. Coating of coverslips.
  a. Place the sample mounts with attached coverslips into the sputter coater.
  b. Start a coating cycle according to the instructions for the device used.

### Step VI: Scanning electron microscope observation

**Timing: 4 h**

The aim of this step is to utilize a scanning electron microscope to observe the diatoms and their associated bacteria, revealing the cellular structure and size, as well as the interaction between bacteria and diatoms.

12. Observation of samples.
  a. Place the sample mount in the scanning electron microscope chamber.
  b. Set the microscope’s acceleration voltage to 5 kV or so to obtain a signal that reflects the surface details of the sample.
  c. Set the electron beam intensity to a value allowing suitable resolution for the magnification used.
  d. Adjust the working distance to take advantage of the resolving power of the detector used. Preferably, use a secondary electrons detector, as the samples are mainly composed of light elements. Adjust the image acquisition speed to avoid irradiation damage to the sample.
  e. Move the microscope stage to select the correct position and adjust focus and astigmatism as well as contrast and brightness. Take images of diatoms and their associated bacteria or measure them.

***Note:*** Once the SEM imaging session has been completed, the samples can be stored for later observation. Storage should be in a box with a lid, to prevent dust from depositing on the surface of the samples, at room temperature and in the dark, to avoid premature degradation of the samples.

To mitigate charging effects, it is important to apply a layer of a conductive material, such as gold or carbon, to the diatom sample as described above. Additionally, one can use lower accelerating voltage to minimize charging artifacts by reducing the interaction volume of the electron beam.

**CRITICAL:** Prolonged or repeated observation of the same sample can lead to progressive degradation. This is because the electron beam from the scanning electron microscope pulls electrons from the surface of the sample, which can degrade it. Additionally, sample storage times can vary depending on the storage conditions. However, the coverslip contains enough cells, offering the option to choose another sample for observation.

## Expected outcomes

**Figure 3:**
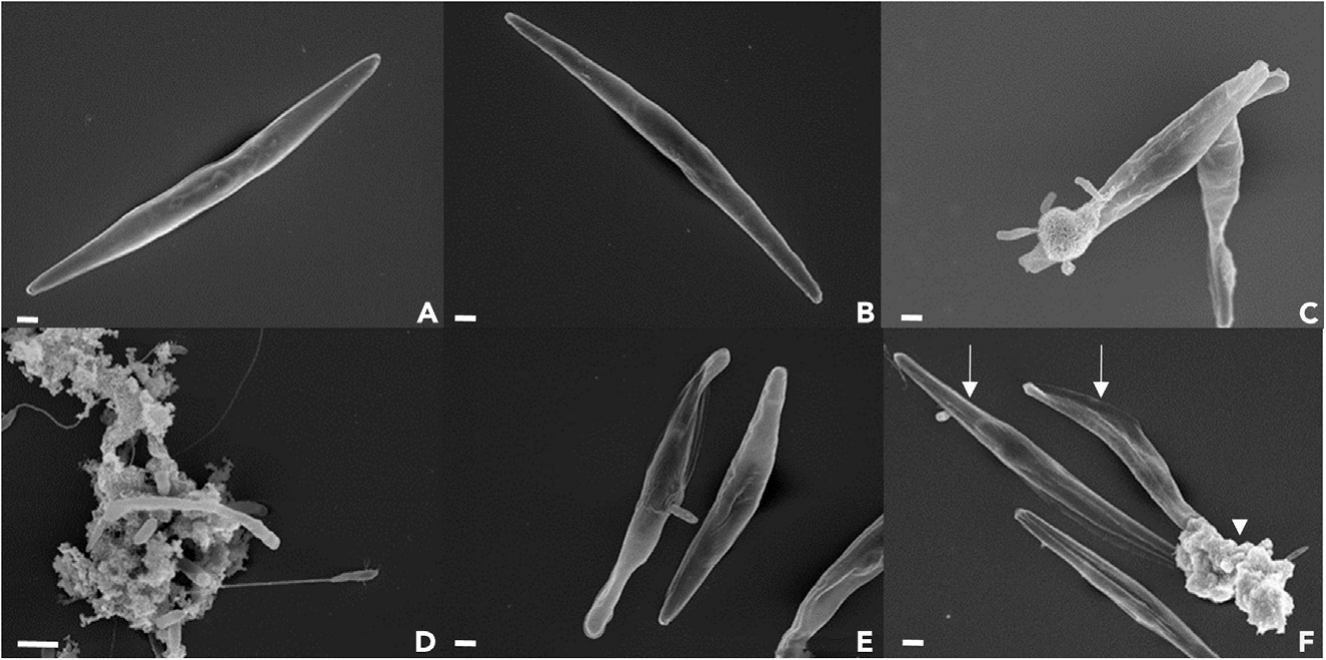
Scanning electron micrographs of *Phaeodactylum tricornutum*, accession 11, and its associated bacteria (A and B) Two individual *P. tricornutum* cells devoid of bacteria. (C) A group of bacteria at the tip of a diatom cell. (D) Different bacteria within a biofilm. (E) A bacterium attached to a diatom cell. (F) Two diatom cells attached to a consortia of bacteria and its biofilm. Diatom cells are marked with arrows, and bacterial consortia are indicated by arrowheads. Scale bar: 1 μm.

This protocol makes it possible to fix *Phaeodactylum tricornutum* cells and associated bacteria while preserving the cell structure. The cells can thus be observed in a state close to their living state (Figure 3).

## Limitations

The protocol has been developed and validated for *Phaeodactylum tricornutum*. However, it may need adjustments when applied to other microalgae species or diatoms. This is particularly important for diatoms, as *P. tricornutum* has minimal silica content, while some other diatoms can be heavily silicified. It is worth noting that silica generally preserves cell integrity, so using a diatom with higher silicification might result in an even smoother process. In conclusion, minor adjustments may be necessary to accommodate different species but this protocol serves as a robust foundation.

## Troubleshooting

### Problem 1: Diatom cell rupture

Diatoms can burst when the cells are at too advanced stage of growth, leading to cell fragility.

**Figure 4:**
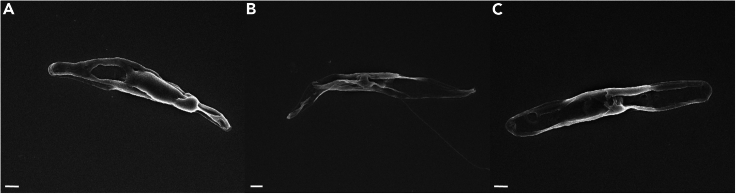
Scanning electron micrographs of damaged *Phaeodactylum tricornutum* cells during fixation and sample preparation for scanning electron microscopy (A–C) Three *P. tricornutum* cells with damage to the cell structure. Scale bar: 1 μm.

Variations in osmotic pressure between the diatom and the different media used for sample preparation, as well as centrifugation time and speed, can cause damage to the cell structure (Figure 4).

### Potential solution

• To prevent the diatoms from bursting, you need to make sure that, at the time of attachment; they are in the middle of their exponential growth phase.
• Reducing centrifugation time or speed also helps to avoid excessive damage to the cell structure of diatoms and associated bacteria. The time and speed described in this study are optimized for *P. tricornutum.*

### Problem 2: Artifact created by contamination on sample surface

**Figure 5:**
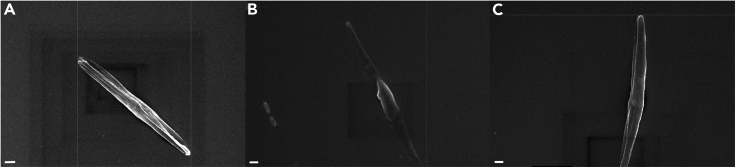
Scanning electron micrographs of *Phaeodactylum tricornutum*, accession 11, and its associated bacteria Examples of images are shown in A, B, and C. Presence of dark square dark artifacts due to surface contamination of samples with ethanol. Scale bar: 1 μm.

When observing freshly prepared samples, artifacts (dark squares) may appear in the images. These artifacts are due to ethanol residues at the sample surface. (Figure 5) They appear when the focused electron beam has scanned the same area over a period of time.

### Potential solution

To address these artifacts, plasma cleaning of the samples is required. This process removes organic contaminants to enhance image resolution and contrast.^15^

## Resource availability

### Lead contact

Further information and requests for resources and reagents should be directed to and will be fulfilled by the lead contact, Dr. Leïla Tirichine (tirichine-l@univ-nantes.fr).

### Technical contact

For technical questions, please contact, Dr. Leïla Tirichine (tirichine-l@univ-nantes.fr).

### Materials availability

This protocol did not generate any new material.

### Data and code availability

This study did not generate a new dataset or code.

## Acknowledgments

We are grateful to the PLASSMAT platform at IMN, Nantes Université for enabling us to use their equipment (Critical Point Dryer, Sputter coater, Scanning Electron Microscope). L.T. acknowledges support from the region of Pays de la Loire (ConnecTalent EPIALG project), Epicycle ANR project (ANR-19-CE20- 0028-02), and μAlgaNIF France-Japan International Research Project.

## Author contributions

L.T. conceived and supervised the study. L.T. and A.T. designed the study. M.M. did the experiment. N.S. supervised critical point drying, metallization, and SEM images acquisition. L.T. obtained the funding. M.M. and L.T. wrote the manuscript. All authors read and approved the manuscript.

## Declaration of interests

The authors declare no competing interests.
